# Solubility and Dissolution Enhancement of Dexibuprofen with Hydroxypropylbetacyclodextrin (HPβCD) and Poloxamers (188/407) Inclusion Complexes: Preparation and In Vitro Characterization

**DOI:** 10.3390/polym14030579

**Published:** 2022-01-31

**Authors:** Rabia Munir, Abdul Hadi, Salah-ud-Din Khan, Sajid Asghar, Muhammad Irfan, Ikram Ullah Khan, Misbah Hameed, Sana Inam, Nayyer Islam, Shahzadi Filza Hassan, Memoona Ishtiaq, Pervaiz Akhtar Shah, Muhammad Shahid Iqbal, Haroon Khalid Syed, Ahmed Khames, Mohammad A. S. Abourehab

**Affiliations:** 1 Department of Pharmaceutics, Faculty of Pharmaceutical Sciences, Government College University, Faisalabad 38000, Pakistan; rabiamunir_786@yahoo.com (R.M.); sajidasghar@gcuf.edu.pk (S.A.); manipharma1@gmail.com (M.I.); ikramglt@gmail.com (I.U.K.); drsanainam@gmail.com (S.I.); nayyerislam1@gmail.com (N.I.); fizahassan32@gmail.com (S.F.H.); memoona62@yahoo.com (M.I.); 2 Department of Medicine, Xian Jiaotong University China, Xian 710000, China; hadi_bargatt@hotmail.com; 3 Department of Biochemistry, College of Medicine, Imam Mohammad Ibn Saud Islamic University (IMSIU), Riyadh 11432, Saudi Arabia; sdikhan@imamu.edu.sa; 4 Institute of Pharmacy, Faculty of Pharmaceutical and Allied Health Sciences, Lahore College for Women University, Lahore 54000, Pakistan; misbahmajid1@gmail.com; 5 Department of Pharmacy, The University of Lahore, Lahore 54000, Pakistan; 6 University College of Pharmacy, University of the Punjab, Lahore 54590, Pakistan; pashah6512@yahoo.com; 7 Department of Clinical Pharmacy, College of Pharmacy, Prince Sattam bin Abdulaziz University, Al-Kharj 11942, Saudi Arabia; m.javed@psau.edu.sa; 8 Department of Pharmaceutics and Industrial Pharmacy, College of Pharmacy, Taif University, P.O. Box 11099, Taif 21944, Saudi Arabia; a.khamies@tu.edu.sa; 9 Department of Pharmaceutics, Faculty of Pharmacy, Umm Al-Qura University, Makkah 21955, Saudi Arabia; maabourehab@uqu.edu.sa; 10 Department of Pharmaceutics, Faculty of Pharmacy, Minia University, Minia 61519, Egypt

**Keywords:** dexibuprofen, HPβCD, PXM, inclusion complex

## Abstract

The objective of this study was to improve the dissolution and solubility of dexibuprofen (DEX) using hydroxypropyl beta cyclodextrin (HPβCD) inclusion complexes and also to evaluate the effect of presence of hydrophilic polymers on solubilization efficiency of HPβCD. Three different methods (physical trituration, kneading and solvent evaporation) were used to prepare binary inclusion complexes at various drug-to-cyclodextrin weight ratios. An increase in solubility and drug release was observed with the kneading (KN) method at a DEX/HPβCD (1:4) weight ratio. The addition of hydrophilic polymers poloxamer-188 (PXM-188) and poloxamer-407 (PXM-407) at 2.5, 5.0, 10.0 and 20% *w/w* enhanced the complexation efficiency and solubility of DEX/HPβCD significantly. Fourier-transform infrared (FTIR) analysis revealed that DEX was successfully incorporated into the cyclodextrin cavity. Differential scanning calorimetry (DSC) and X-ray diffractometry (XRD) revealed less crystallinity of the drug and its entrapment in the cyclodextrin molecular cage. The addition of PXM-188 or PXM-407 reduced the strength of the DEX endothermic peak. With the addition of hydrophilic polymers, sharp and intense peaks of DEX disappeared. Finally, it was concluded that PXM-188 at a weight ratio of 10.0% *w/w* was the best candidate for improving solubility, stability and release rate of DEX.

## 1. Introduction

**Figure 1:**
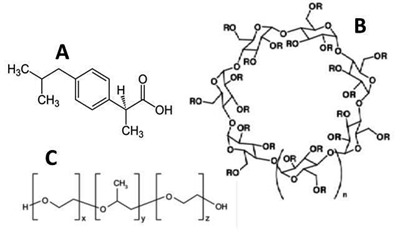
Chemical structure of (**A**) DEX, (**B**) HPβCD and (**C**) poloxamers. Note: x, y and z are hydrophilic ethyleneoxide (EO), hydrophobic propyleneoxide (PO) and hydrophilic (EO) units.

To design safe and effective dosage form, drug solubility is the most important parameter among all others. In recent years, the number of hydrophobic drug molecules used in the treatment of different diseases has been comparatively higher, and their absorption and biological activity all depend on aqueous solubility. Poor water solubility and dissolution are key challenges for BCS class II (low solubility and high permeability) drugs [1,2,3,4]. To address the limitations of BCS Class II drugs, several methods have been developed, including salt formation, lipid-based formulations, particle size reduction, solid dispersions, complexation with cyclodextrins or its derivatives [5,6,7,8,9] and supercritical antisolvent co-precipitation [10]. Cyclodextrins (CDs) inclusion complexation is one of the several methods available for improving the solubility of drug molecules. The most important function of CDs is their ability to alter the physical and chemical properties of guest molecule within their inner cavity and formation of effective inclusion complex [7,8]. Inclusion complexation may enhance the bioavailability and efficacy of the drugs, and this may ultimately reduce the dose frequency [9,10,11]. A hydroxy-alkyl-cyclodextrin derivative of cyclodextrin, i.e., hydroxypropyl-β-cyclodextrin (HPβCD), has an amorphous structure, more aqueous solubility and a lower toxicity profile, and it has been effectively used in pharmaceutical formulations [12]. HPβCD (Figure 1B) can increase the drug release, leading to the improvement of the drug absorption across biological barriers. Furthermore, the amorphous structure of HPβCD is significant in reducing polymorphic transition and crystallization of poor water-soluble drugs, thus ultimately enhancing the drug solubility and oral bioavailability.

The use of CD is restricted because guest molecule (drug) assembling, whether entirely or partially, within the cavity of the CD molecule is perilous for the guest molecule [7]. The addition of hydrophilic polymers was found to improve the solubilization and complexation efficiency of cyclodextrins [13].

For example, poloxamers (1C) are added to the formulation to improve the solubility and dissolution behavior of weakly water-soluble active pharmaceutical ingredients (APIs) in solid dosage forms [14,15]. PXM-188 improves the dissolution profile significantly by impeding the drug crystalline state, resulting in an amorphous form of the drug [16]. 

In previous works in the literature, it was revealed that ibuprofen (IBF) and β-cyclodextrin (βCD) binary inclusion complexes were prepared at 2:3 weight ratio by various methods, such as physical mixing, co-precipitation–evaporation (CE), co-precipitation–centrifugation (CC), spray-drying (SD) and freeze-drying (FD) [17]. At molar ratios of 1:1 and 2:1 (HPβCD/ibuprofen), electrospun nanofibers were generated to enhance the solubility of ibuprofen [18], and ibuprofen–captisol® (sulfobutylether sodium salt of β-CD) binary inclusion complexes were also prepared to enhance the in vitro dissolution [19]. Mianserin hydrochloride (MIA) toxicity could be decreased by encapsulating the drug inside the beta-cyclodextrin molecules (βCD) [20]. Shuang et al. prepared hydroxypropyl cyclodextrin/difenoconazole inclusion complex (HPβCD/DZ-IC) nanofibers, which showed promise as a novel fast-dissolving pesticide formulation [21]. Perillaldehyde/hydroxypropyl-γ-cyclodextrin inclusion complex nanofiber (PA/HPγCD-IC-NF) was produced by electrospinning. PA ((water immiscible liquid)) can be stored as a solid inclusion compound with enhanced aqueous solubility and improved physicochemical properties [22].

Dexibuprofen (DEX), known as S (+)-ibuprofen, is more effective and has different physicochemical characteristics than racemic ibuprofen. Because of a high concentration of the active S-enantiomer in racemic mixture, DEX is considered to be pharmacologically more active and tolerated than ibuprofen and has a better safety profile. It has poor aqueous solubility, belongs to BCS class II and has a short pharmacological half-life (t1/2) of 1.8 to 3.5 h [23,24]. It shows the erratic bioavailability and variability in absorption. DEX is primarily useful to control mild-to-moderate pain, such as headache, postoperative and dental pain, dysmenorrhea, soft-tissue rheumatism and inflammatory conditions [25,26]. The chemical structure of DEX is presented in Figure 1A. Mixed hydrotropic solubilization [27], nanocrystals [28] and binary complexes with βCD are also reported [29,30] to enhance the solubility of DEX. In the current study, we intended to decrease the DEX limitation by using ternary complexes. To achieve these objectives, we used hydrophilic polymers, poloxamers (Figure 1C). These hydrophilic polymers could ameliorate the solubilizing and complexation efficiency of cyclodextrins [31,32,33].

The purpose of this research work is to investigate the effect of hydrophilic polymers (PXM-188 and PXM-407) on the DEX:HPβCD binary inclusion complex. The binary (DEX: HPβCD) and the ternary inclusion complexes (DEX:HPβCD:PXM) were studied for phase solubility. The solubility and dissolution study of the binary and ternary inclusion complexes was performed to check the effect of ternary substances (PXMs).

All the complexes were characterized by using FTIR, SEM, XRD and DSC.

## 2. Material and Methods

### 2.1. Materials 

DEX reference standard was gifted from SAMI Pharmaceutical (Pvt) LTD., Karachi (Pakistan). Hydroxypropyl β-cyclodextrin (HPβCD) (1380 gm molecular weight, 278 °C melting point and 0.6 molar substitution) was supplied by Roquette (Lestrem, France). Poloxamer-188 (PXM-188) and poloxamer-407 were bought from Sigma-Aldrich (St. Louis, MO, USA). Ethanol 99% (35%) was bought from British Drug Houses (BDH). Distilled water was provided by pharmaceutical lab, faculty of pharmaceutical sciences, GCUF. All solvents and chemicals were of analytical grade.

### 2.2. Methods

#### 2.2.1. Phase Solubility Study

The Higuchi and Connors (1965) method was used to conduct the phase solubility study [34]. Excess quantity of DEX was introduced in 10 mL of molar solutions (5–20 mM) of HPβCD with and without different concentrations of hydrophilic polymers (0, 2.5, 5.0, 10.0 and 20% *w/v*). To maintain equilibrium, the solutions were kept in a shaking water bath (TSSWB15-USA) at 37 °C for 72 h. The samples were filtered after centrifugation at 6000 rpm for 30 min. The quantity of DEX in the samples was measured by using a UV spectrophotometer (CECIL 7400-S, Cambridge, England) at 223.5 nm. The following equations were used to quantify the stability constant (Ks) and complexation efficiency (C.E).

(1) $$Ks=\frac{slope}{\mathrm{So}\left(1-slope\right)}$$

where S_o_ is the equilibrium aqueous solubility of DEX, and the slope is attained by plotting DEX concentration versus HPβCD concentrations with or without polymer.

(2) $$C.E=\frac{slope}{1-slope}$$

#### 2.2.2. Preparation of Binary Inclusion Complexes

HPβCD was used for the preparation of the DEX/cyclodextrin binary inclusion complex at 1:1, 1:2, 1:4 and 1:8 weight (*w/w*) ratios. Three different methods were used to prepare binary inclusion complexes (physical trituration, solvent evaporation and kneading method).

Physical trituration (PT) was performed by properly mixing the weighed amounts of DEX and HPβCD in a mortar and pestle for up to one hour to ensure a uniform mixture. The final powder mixture was then sieved through sieve number 60 and stored in air tight container.

In the solvent evaporation (SE), HβPCD was dissolved in 25 mL water and DEX was in ethanol, followed by thorough mixing of these two solutions with a magnetic stirrer at 50 °C. Rotary evaporator was used to evaporate the solvents. The dry powder material was collected and stored in an air-tight container.

In the kneading (KN) method, the drug (DEX) and polymer (HPβCD) were physically mixed in four different ratios, namely 1:1, 1:2, 1:4 and 1:8 (*w/w*), and kneaded for 1 h in a mortar with ethanol–water (1:1 *v/v*) to obtain a consistency akin to a slurry mass. It was placed at 45 °C in an oven to dry. The resultant mass was crushed and sieved through a sieve with a mesh size of 0.25 mm and kept in an air-tight container for further use. 

#### 2.2.3. Preparation of Ternary Complexes by Kneading Method

Ternary inclusion complexes were prepared by adding the hydrophilic polymer (PXM-188 or PXM-407) at different concentrations (2.5%, 5.0%, 10.0% and 20%) of dry weight (*w/w*) of binary inclusion complexes (having highest solubility). Hydrophilic polymers (PXM-188 or PXM-407) were added to the prepared binary inclusion complex, followed by all other necessary parameters of the abovementioned kneaded method.

#### 2.2.4. Solubility Study 

To carry out the solubility study, the excess amount of prepared binary and ternary inclusion complexes was added into distilled water (10 mL). The flasks were vortexed for three minutes and placed for 72 h at 100 rpm in a shaking water bath (TSSWB15-USA), which was held at 25 ± 2 °C. Then 3 mL of sample was taken and passed through a membrane filter of 0.45 µm. The filtrate (100 µL) was diluted if necessary and analyzed by spectrophotometer (CECIL 7400-S, Cambridge, UK). The experiment was repeated three times.

#### 2.2.5. In Vitro Dissolution Studies

In vitro release studies were performed for DEX and for all prepared binary and ternary inclusion complexes under sink conditions. For this purpose, the USP dissolution device (PTWS 3CE, Pharma test, Hainburg, Germany) apparatus II paddle method (USP XXXII, 2009) was used. Dissolution medium was encompassed on 900 mL of distilled water that was kept at 37 ± 0.5 °C, with paddle rotating at a speed of 100 rpm. The quantity of powder equivalent to 10 mg of the drug (DEX) was put into each vessel of the dissolution apparatus. At programmed time intervals of 5, 10, 20, 30, 40, 50 and 60 min, a sample of 5 mL was taken, followed by a replacement with fresh dissolution media. The sample was filtered through a 0.45 μm membrane filter. A UV spectrophotometer (CECIL 7400-S, Cambridge, England) was used for quantification of the drug.

The dissolution efficiency (DE) was calculated by the trapezoidal method by using the following equation:

(3) $$\mathrm{DE}\left(\%\right)=\frac{\int_{0}^{t}y\;\times \;\mathrm{dt}}{y\;100\;\times \;t}\;\times \;100\%$$

In the abovementioned equation, y is the percentage of dissolved DEX. DE is the area under the dissolution curve among time point t_1_ and t_2_, stated as a percentage of the curve at maximum dissolution, y100, over the same period. It was calculated to quantify the presence of a variety of carriers in inclusion complexation.

#### 2.2.6. Scanning Electron Microscopy (SEM)

Surface morphology of DEX, HPβCD, PXM-188, PXM-407, and binary and ternary inclusion complexes was analyzed by using scanning electron microscopy (SEM) at 2 KeV. To increase the electrical conductivity, samples were stammered in a tinny coating of gold.

#### 2.2.7. Fourier-Transform Infrared Spectroscopy (FTIR)

FTIR analysis gives the possible information of functional groups and structures. In FTIR, the infrared spectra of samples (drug, polymers, and binary and ternary inclusion complexes) were scanned (BRUKER Tensor II-Alpha, Berlin, Germany) from 400 to 4000 cm^−1^ by using the potassium bromide disk. 

#### 2.2.8. Differential Scanning Calorimetry (DSC)

DSC (Universal V4.2E TA Instruments, Newcastle, DE, USA) device was used to test differential scanning calorimetry measurements of DEX, HPβCD, both hydrophilic polymers (PXM-188, PXM-407), and binary and ternary inclusion complexes of DEX. In an aluminum pan, a sample of 3 to 10 mg was measured and crushed. The samples were scanned under a nitrogen purge of 20 mL/min at a steady rate of 10 °C per minute over a range of 20–400 °C. Empty aluminum pan was used as a reference.

#### 2.2.9. Powder X-ray Diffraction Study (XRD)

The physical form of DEX, HPβCD, PXM-188, PXM-407, and binary and ternary inclusion complexes was analyzed by X-ray diffractometer (XRD) (Bruker D-8 Advance, Berlin, Germany), using graphite monochromator with Cu-Kα radiation. X-ray tube was run at 40 kV and 30 mA current. Samples were analyzed at a speed of 5° per minute with increments of 0.05° over a range (2θ) to scan from 5° to 80°.

### 2.3. Statistical Analysis

The data were statistically analyzed by using SPSS (version 20) and expressed as mean and standard deviation (±SD). One-way analysis of variance (ANOVA) was used to evaluate solubility data and dissolution efficiency (DE). A post hoc Tukey HSD test was used to calculate statistically significant difference. The difference was definite statistically significant when the *p* < 0.05 value was used.

## 3. Results and Discussion

### 3.1. Phase Solubility Study

**Figure 2:**
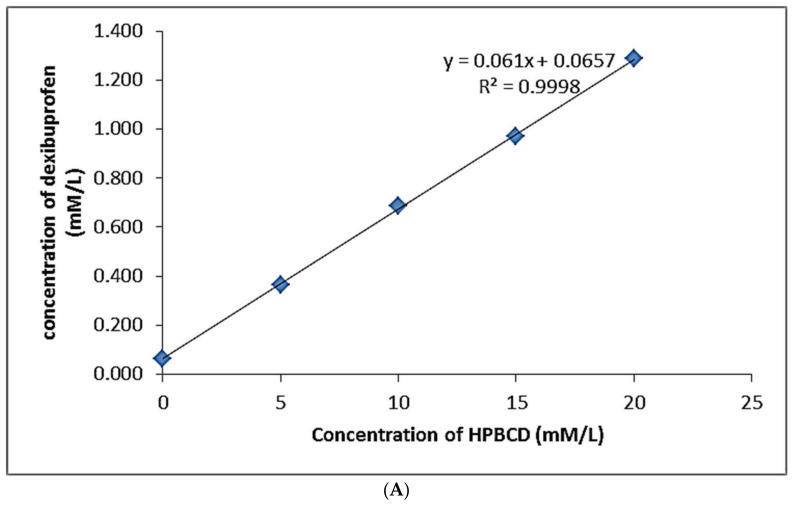

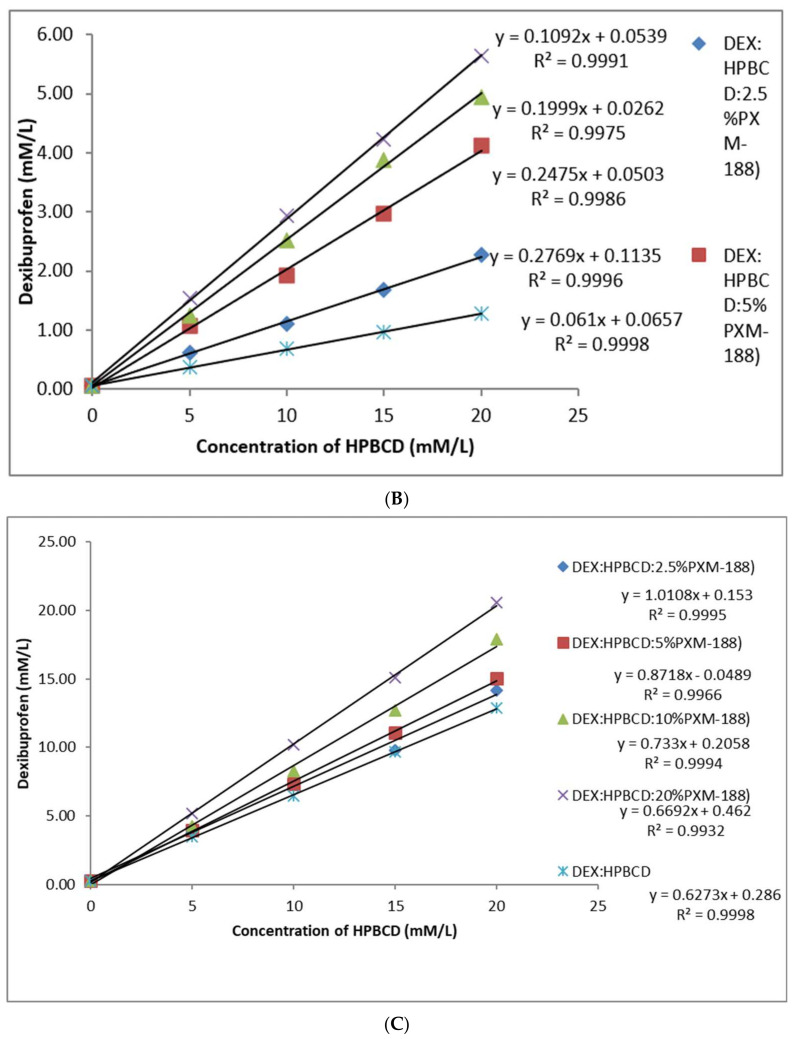
(**A**) Phase solubility diagram of DEX in aqueous solution of HPβCD mean ± SD, *n* = 3. (**B**) Phase solubility diagram of DEX in aqueous solution of HPβCD with/without PXM−188. (**C**) Phase solubility diagram of DEX in aqueous solution of HPβCD with/without PXM−407.

Figure 2A–C illustrates the phase-solubility graphs of binary and ternary inclusion complexes. This study revealed that, in the case of the binary inclusion complex, the increase in the polymer (HPβCD) concentration contributed to the comparative increase in drug solubility [18,34]. This can be categorized as the A_L_ type of solubility diagram and formation of the soluble complex. 

An indication of the process of transfer of DEX from water to the aqueous solution of HPβCD was obtained from the values of Gibbs free-energy change. The Gibbs free energy (ΔG^0^) of DEX from pure water to the aqueous solution of cyclodextrin was calculated from the following equation:

ΔG^0^ = −2.303 RT log [S_0_/Ss] (4)

where S_0_/Ss is the ratio of solubility of drug in the absence and in the presence of cyclodextrin.

It was negative for all the concentrations of HPβCD, showing that DEX solubilization was spontaneous. ΔG^0^ was decreased as the HPβCD concentration increased and the reaction became more favorable [35]. This indicated the more stability and formation of stable inclusion complex. Results are presented in Table 1.

PXM-188 produced the highest slope value and drug solubility [36]. The value of the stability constant for both the binary and ternary complexes was from 200 to 5000 M^−1^ [37]. From their stability constant values (Table 2), it was clear that ternary inclusion complexation has the ability to improve the DEX solubility more than binary inclusion complex. The phase-solubility study indicated that the ternary system was more effective on binary in the presence of the ternary component [38].

### 3.2. Solubility Study of Ternary Complexes

Table 3 presented the solubility data for DEX and the binary and ternary inclusion complexes of DEX. It was determined that, with the binary inclusion complex, the solubility was improved significantly from 0.0617 mg/mL (pure DEX) to 18.23 mg/mL (DEX:HPβCD 1:8). The KN method gave the highest solubility (18.23 mg/mL) at 1:8 *w/w* ratio, followed by PT (11.55 mg/mL) and finally with the SE method (5.04 mg/mL). The concentration of HPβCD used to solubilize DEX was important, and it was noted that, in all three methods, the solubility of DEX was increased. In the case of the KN method, when the concentration of HPβCD was increased from 1:4 (17.13 mg/mL) to 1:8 (18.23 mg/mL), no significant improvement in the solubility of DEX (only with minute increase) was shown, so we excluded the 1:8 from further analysis, since, at this point, the solubility was not increased accordingly with the high polymer concentration. 

The incorporation of the hydrophilic polymer within the binary inclusion complex significantly increased the solubility of DEX (42.66 mg/mL). The literature has also illustrated that poloxamer incorporation can increase the solubility of less-soluble drugs, such as insulin and indomethacin. When PXM-407 (22.5% *w/w*) was incorporated with piroxicam, the solubility of piroxicam was enhanced up to 11-fold. The solubility of nifedipine with PXM-407 (4% *w/v*) was also improved up to 27-fold and led to complete dissolution [39]. The same results were found in this solubility study: as the concentration of the polymer was increased from 2.5 to 20% (*w/w*) of dry weight of the binary inclusion complex, the solubility was also increased. In the case of the ternary inclusion complexation, PXM-188 presented the maximum DEX solubility enhancement effect as compared to PXM-407. The increase in solubility with the increase in poloxamer concentration indicates that PXM-188 has solvent properties for the drug. The interfacial tension between the drug and the solubility media was reduced by PXM-188 [40].

The ternary inclusion complexes had significantly increased the solubility, because of the lower crystallinity of the drug and greater amorphousness of the polymer [34,35]. On the basis of solubility studies with different concentrations of both polymers, it was found that PXM-188 was the best because of more solubility of DEX (42.66 mg/mL), as compared to PXM-407 (34.88 mg/mL) at comparable ratio (20%). Thus, using a mixture of HPβCD/hydrophilic polymers rather than one of them alone improved the solubility of the drug moiety more effectively, and among the ternary complexes, DEX:HPβCD:PXM-188 (1:4:10%) was found to be the best, because of the significant increase in solubility as compared to the other ratio. DEX:HPβCD:PXM-188 (1:4:20%) had a non-significant increase in solubility as compared to DEX:HPβCD:PXM-188 (1:4:10%), even by the 2-fold increase in the percentage of PXM-188. 

The maximum solubility was recorded for the DEX/HPβCD/PXM-188 ((1:4:20%) ternary complex (42.54 mg/mL) at approximately 688 times that of pure drug solubility (0.062 mg/mL) and was considered statistically significant (*p* < 0.05). A post hoc Tukey HSD test was used to analyze the data.

The ternary complex was better than the binary systems in terms of complexation efficiency and solubility. The polymer interacted with drug/CD complexes through the outer surface of CD molecules, producing aggregates capable of solubilizing drug and other hydrophobic compounds [41,42].

### 3.3. In Vitro Dissolution Studies

The encapsulation efficiency of all prepared complexes was determined, and it was found to be more than 97% DEX entrapment. 

**Figure 3:**
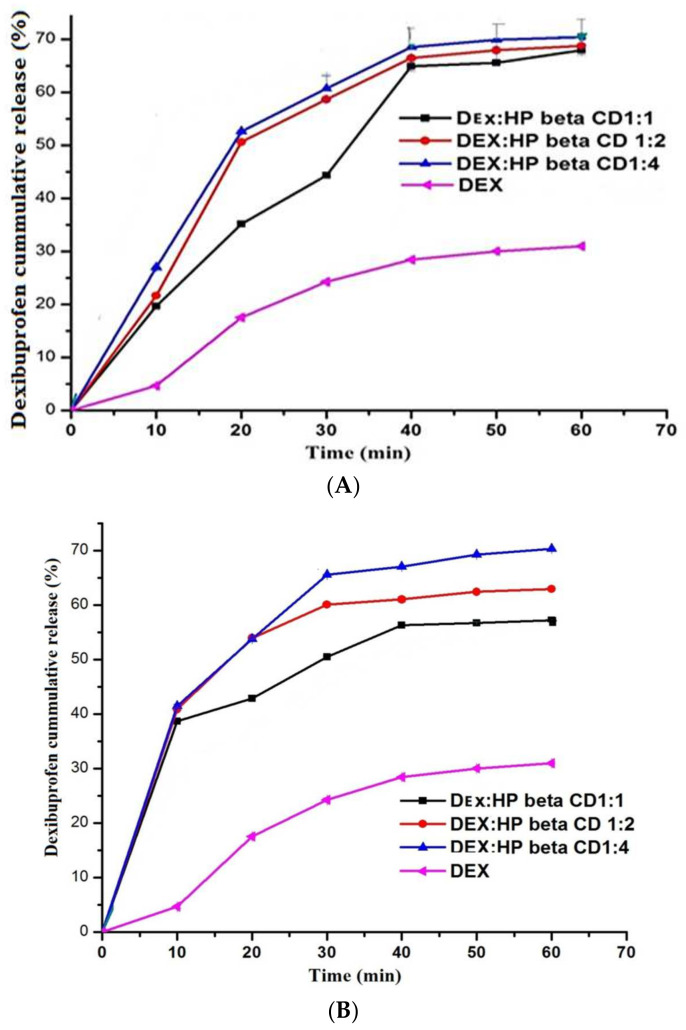

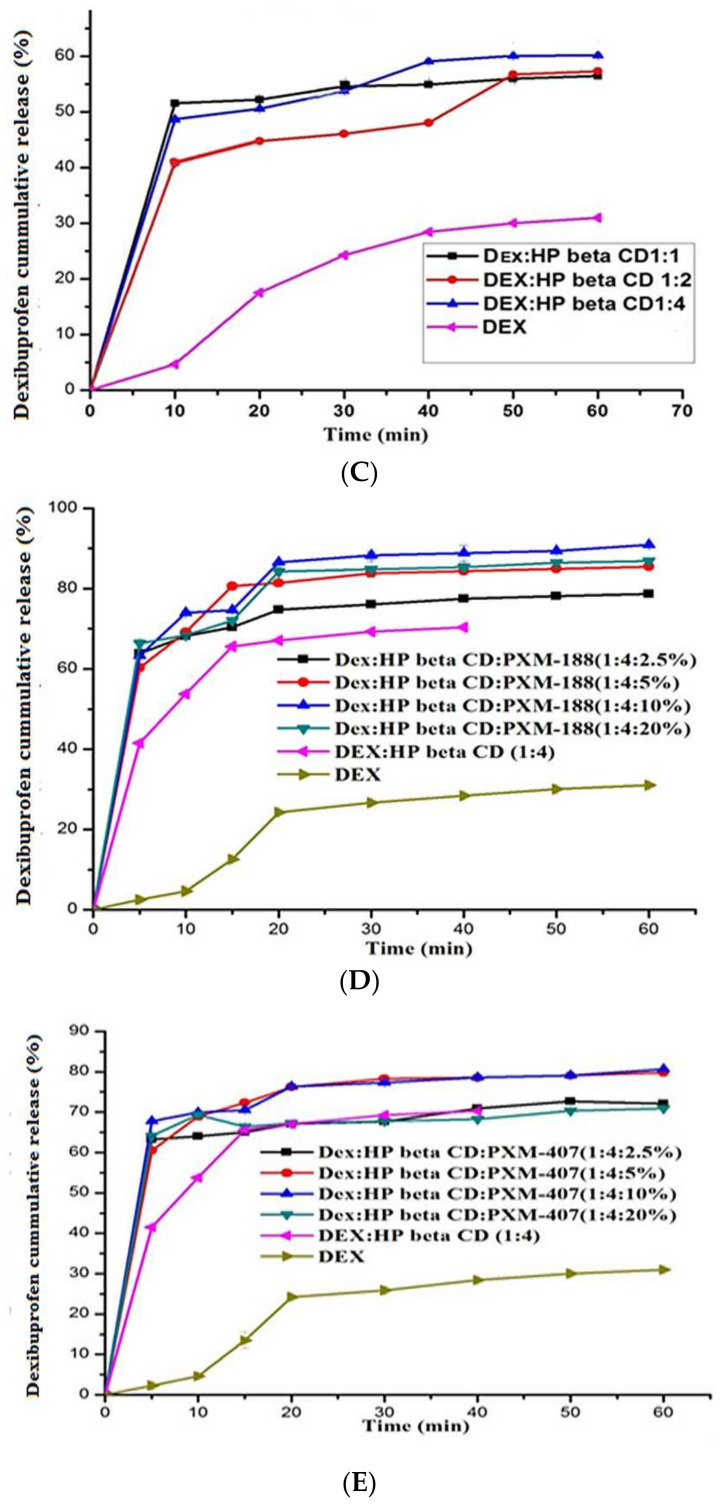
(**A**) Dissolution profiles of DEX and DEX–HPβCD inclusion complexes by PT method Mean ± SD, *n* = 3. (**B**) Dissolution profiles of DEX and DEX–HPβCD inclusion complexes by KN Mean ± SD, *n* = 3. (**C**) Dissolution profiles of DEX and DEX–HPβCD inclusion complexes SE method. Mean ± SD, *n* = 3. (**D**) Dissolution profiles of DEX, binary system and DEX:HPβCD ternary systems with PXM-188. Mean ± SD, *n* = 3. (**E**) Dissolution profiles of DEX, binary system DEX:HPβCD and ternary systems with PXM-407. Mean ± SD, *n* = 3.

The % cumulative drug release of DEX and the binary and ternary complexes is presented in Figure 3A–E. Pure DEX was released with a minimum release rate of about 30% because of its low aqueous solubility. After an hour, more than 50% drug release was observed in the binary inclusion complexes. It was found that the binary inclusion complex prepared by using the KN method at a DEX:HPβCD (1:4) ratio had a release rate that was up to 72% and was different significantly from that of pure DEX. 

The release rate was increased because of enhancement of solubility [42,43]. DEX’s solubility was improved by partial entrapment of hydrophilic polymer in the CDs complex, allowing for the fastest release of the drug. The highest release was observed by PXM-188 at 10% *w/w*. Because the crystallinity of the drug was reduced, the dissolution process required less energy, and the drug was dispersed uniformly [39]. The drug release was decreased or slowed down when the % amount of PXM-188 and PXM-407 was increased from 10% to 20%. This was because of the drawback of PXM having low mechanical power, which causes the polymer to erode quickly. Poloxamer has also been shown to have the capacity to convert from a less viscous solution to a gel at room temperature when used in 18% or more [44]. At a high molecular weight and higher proportion of hydrophobic polyoxypropylene segment (lower HLB value), PXM-407 was less effective than PXM-188. When higher poloxamer ratios were used in the inclusion complex, the intrinsic dissolution rate was decreased as compared to the lower ratios. This impact was especially noticeable in the case of PXM- 407. On the other hand, greater amounts of poloxamers are thought to improve gel-layer formation in water, which acts as a diffusion barrier and delays drug release [45,46].

HPβCD, along with PXM-188, increased the dissolution of hydrophobic drug in the ternary inclusion complexes prepared by using the KN method. When comparing the binary and ternary complexes, the amorphousness (lack of crystallinity), solubility, dispersibility and small particle size are all regarded as important factors for dissolution. Furthermore, the mixing of the hydrophobic drug with a hydrophilic carrier (PXM-188) may increase the surface area for drug release and interfacial tension between the hydrophobic drug; moreover, the dissolution media was decreased, and drug release was increased [41]. The binary inclusion complex showed the highest drug release within 60 min, at between 50 and 72%, while the ternary complex showed the highest release, at 90%. For the ternary inclusion complex, 1:4 (10.0% PMX-188) was found to be the best not only on the basis of solubility but also because of the high dissolution rate. Table 4 shows the findings for dissolution efficiency (DE). A one-way ANOVA test was used to statistically measure the dissolution efficiency (DE) (post hoc Tukey HSD test). Thus, it was concluded that DEX inclusion complexes showed high rates of in vitro dissolution and DE, and they have a statistically significant difference from those of pure drug (DEX).

### 3.4. Scanning Electron Microscopy (SEM)

**Figure 4:**
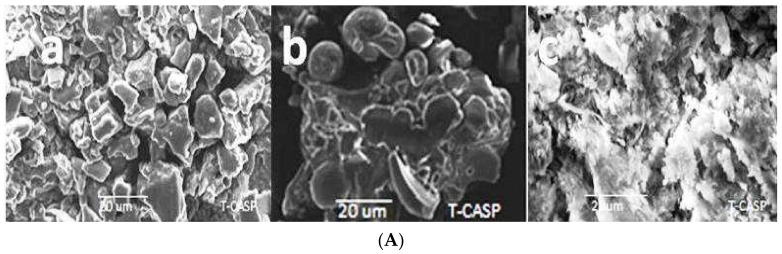

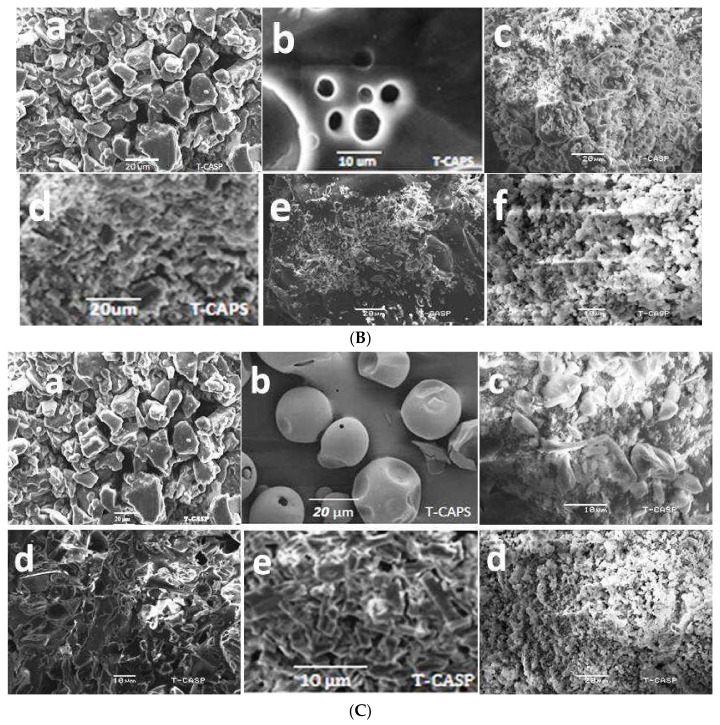
(**A**) SEM pictures of (a) DEX, (b)HPβCD and (c) 1:4 DEX:HPβCD. (**B**) SEM pictures of (a) DEX, (b) PXM-188, (c), 1:4 DEX:HPβCD:PXM-188 2.5%, (d) 1:4 DEX:HPβCD:PXM-188 5.0%, (e) 1:4 DEX:HPβCD:PXM-188 10% and (f) 1:4 DEX:HPβCD:PXM-188 20%. (**C**) SEM pictures of (a) DEX, (b) PXM-407, (c) 1:4 DEX:HPβCD:PXM-407 2.5%, (d) 1:4 DEX:HPβCD:PXM-407 5.0%, (e) 1:4 DEX:HPβCD:PXM-407 10% and (f) 1:4 DEX:HPβCD:PXM-407 20%.

The SEM images demonstrate the morphological distinctions between the individual components and inclusion complexes. DEX had irregularly formed particles/crystals of no discernible form [37]. HPβCD is in amorphous form with indistinguishable shapes [45]. The DEX-HPβCD SEM images exposed small particles with an inclination to accumulate, indicating the presence of less crystallinity and a single component in the complex (Figure 4A). The KN method transformed the drug (DEX) from being crystalline to a less-crystalline form and increased the drug solubility and dissolution.

PXM-188 had a vast globular shape [46,47,48,49], and PXM-407 also had globular spiral shape, but it does not have as fine a shape as PXM-188 (Figure 4B,C). The SEM images of ternary inclusion complexes showed that the crystallinity was reduced because of ternary inclusion complex formation and uniform drug dispersion within carrier molecules [50]. HPβCD, along with PXM-188, developed an amorphous ternary complex more than PXM-407. In the case of PXM-188, the ternary inclusion complex seemed to be mixture or in an aggregated form. The SEM images of ternary inclusion complexes revealed very small particles with a greater tendency to penetrate and diffuse. The SEM images illustrated that the basic composition of components was extinct and the original pure compounds could not be identified.

### 3.5. Fourier-Transform Infrared Spectroscopy (FTIR)

**Figure 5:**
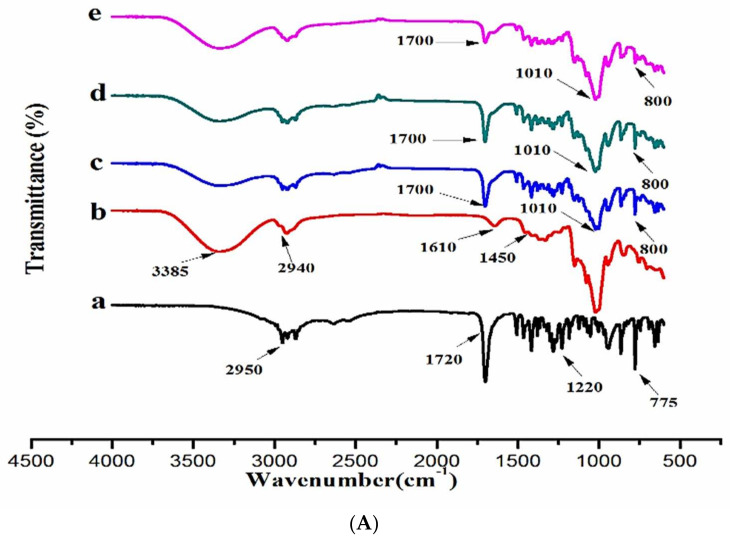

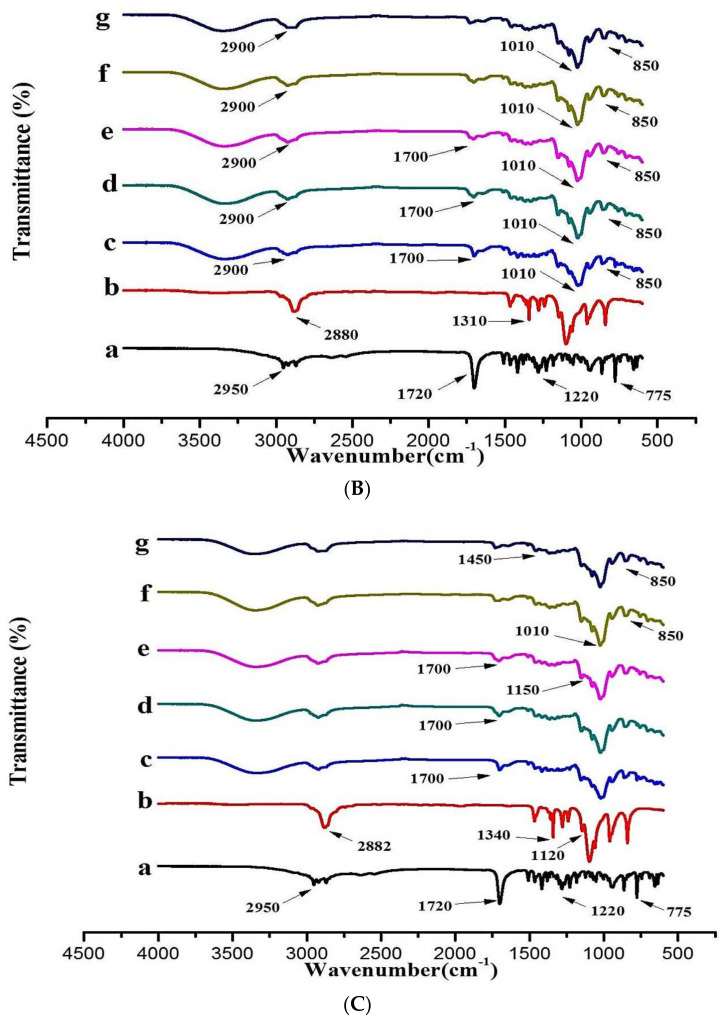
(**A**) FTIR spectrum of (a) DEX, (b) HPβCD, (c) DEX/HPβCD (1:1), (d) DEX/HPβCD (1:2) and (e) DEX/HPβCD (1:4) prepared by kneading method. (**B**) FTIR spectrum of (a) DEX, (b) PXM−188, (c) 1:4 DEX:HPβCD, (d) 1:4 DEX:HPβCD:PXM−188 2.5%, (e) 1:4 DEX:HPβCD:PXM−188 5.0%, (f) 1:4 DEX:HPβCD:PXM−188 10% and (g) 1:4 DEX:HPβCD:PXM−188 20%. (**C**) FTIR spectrum of (a) DEX, (b) PXM−407, (c), 1:4 DEX:HPβCD, (d) 1:4 DEX:HPβCD:PXM−407 2.5%, (e) 1:4 DEX:HPβCD:PXM−407 5.0%, (f) 1:4 DEX:HPβCD:PXM−407 10% and (f) 1:4 DEX:HPβCD:PXM−407 20%.

Figure 5A–C shows the FTIR spectra of DEX, PXM-188, and binary (DEX: HPβCD) and ternary complexes of DEX (DEX:HPβCD:PXM-188). FTIR bands of DEX revealed typical O–H extending at 3052 cm^−1^ and CH_3_ stretching at a length of 2950 cm^−1^, and the C=O (free acid carbonyl) peak appeared at a distance of 1720 cm^−1^; the peak at 775 cm^−1^ is because of the vibration of CH_2_ group, and a sharp peak at 1220 cm^−1^ was due to C-C stretching. These are the most important peaks of the FTIR spectrum of DEX.

HPβCD showed sharp and wide bands between 3300 and 3400 cm^−1^, corresponding to stretching vibrations of -OH groups, due to intermolecular hydrogen bonds. The peak at 2950 cm^−1^ was assigned to the stretching vibration of aliphatic C-H group, a band at 1610 cm^−1^ is indicative of the twist vibration of O-H group and C-O stretching vibration bands were situated between 3300 and 3400 cm^−1^ [51]. The absorption peak of α-type glycosidic bond was obtained at 800 cm^−1^, indicating that CDs were produced by glucopyranose units by α-1,4-glycosidic bond. The absorption peak at 3400 cm^−1^ corresponds to the stretching vibration of O-H. The absorption peak was broad and intense because of the intramolecular hydrogen bond’s drop in the bond-force constant [52]. The principal absorption peaks in the spectrum of PXM-188 were found at 2880 cm^−1^ (C-H stretch aliphatic), 1310 cm^−1^ (in plane O-H bend) and 1111 cm^−1^ (C–O stretch). PXM-407 had absorption peaks at 2882 cm^−1^ (C–H stretch aliphatic), 1340 cm^−1^ (in-plane O–H bend) and 1120 cm^−1^ in its FTIR spectrum. (C–O stretch), and they were uniform for all ternary systems (DEX:HPβCD:PXM-407). The bands remained unchanged in the ternary complex of DEX:HPβCD:PXM-188. 

The peak pattern of the pure drug was not changed considerably, and this shows that the components had no chemical interactions [53] in both the binary and ternary complexes. However, in the case of the binary inclusion complex DEX:HPβCD at a 1:4 weight ratio, we perceived the shifting of peaks and complexity of the inclusion complex as compared to individual components. The kneading method reduced the sharpness of the peaks [54]. The spectra of ternary inclusion complexes are mostly similar to the spectra of individual components, although there are some very small changes that may suggest the presence of intermolecular interactions between drug and PXM. When the PXM ratio in the complex is increased, the C-H stretching vibrations change from 2880 to 2900 cm^1^. These minor alterations in FTIR spectra are most likely the consequence of hydrogen-bond formation between DEX and PXM. However, no further peak was observed in the inclusion complexes’ spectra, demonstrating the absence of any chemical interaction between DEX and poloxamers.

It was observed that there are no interactions between DEX:HPβCD:PXM-188; no new peaks were formed, but were the peaks were slightly shifted, and the intensity was reduced in the case of ternary complexation. The reduction in the peak intensity might be due to the Vander Waals forces and hydrogen bonding between the DEX and hydrophilic polymers, as this could give the increase in dissolution rate of drug [55].

### 3.6. Differential Scanning Calorimetry (DSC)

**Figure 6:**
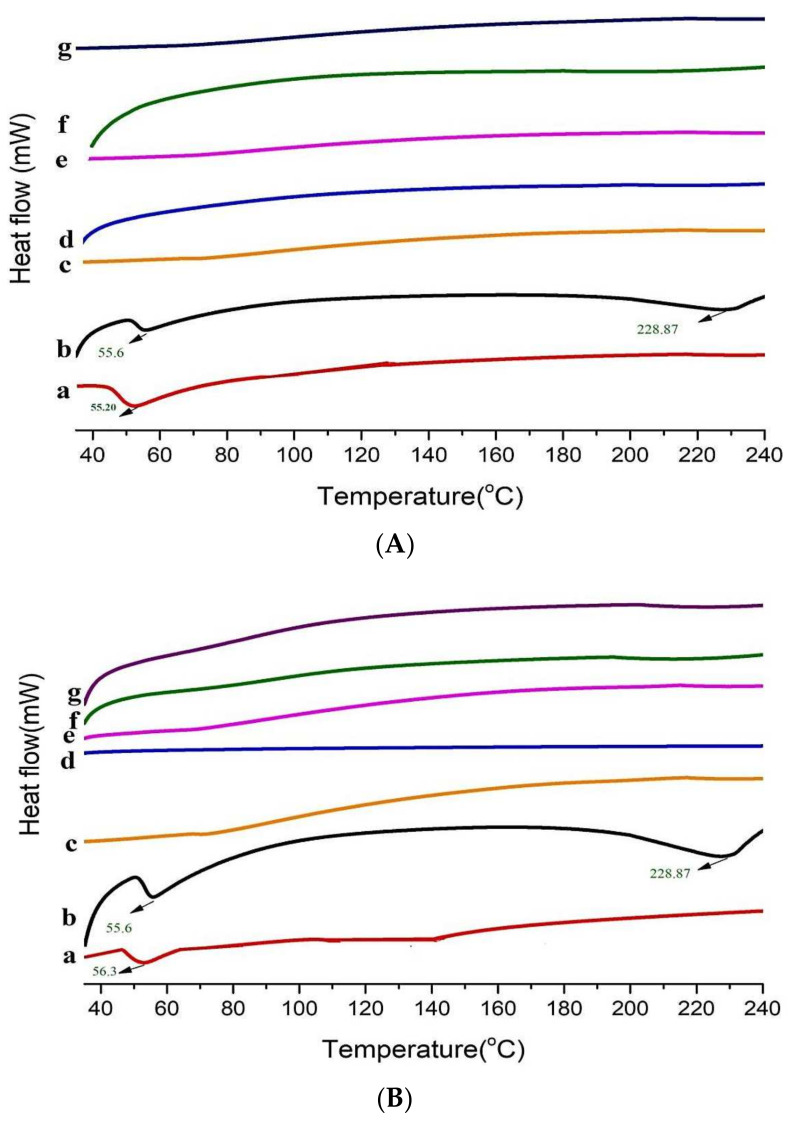
(**A**) DSC thermo-grams of (a) DEX, (b) PXM−188, (c), 1:4 DEX:HPβCD (d) 1:4 DEX:HPβCD:PXM−188 2.5%, (e) 1:4 DEX:HPβCD:PXM-188 5.0%, (f) 1:4 DEX:HPβCD:PXM−188 10% and (g) DEX:HPβCD:PXM−188 20%. (**B**) DSC thermo-grams of (a) DEX, (b) PXM−407, (c), 1:4 DEX:HPβCD, (d) 1:4 DEX:HPβCD:PXM−407 2.5%, (e) 1:4 DEX:HPβCD:PXM−407 5.0%, (f) 1:4 DEX:HPβCD:PXM4−07 10% and (g) 1:4 DEX:HPβCD:PXM−407 20%.

DSC is used to analyze the thermal properties of drug (DEX), polymers (HPβCD, PXM-188 and PXM-407), and cyclodextrin binary and ternary inclusion complexes (DEX:HPβCD, DEX:HPβCD:PXM-188 and DEX:HPβCD:PXM-407). This analysis may provide knowledge about the physicochemical status of the guest molecule within the cavity of cyclodextrin (host). Absence or moving of the endothermic peak of molecules may provide the information about changes in the crystal lattice, melting point, boiling point, or sublimation [41]. The DEX DSC profile revealed a pronounced endothermic peak, consistent with its melting transition temperature at *T_max_* of 55.6 °C. In Figure 6A, an endothermic peak of PXM-188 was at 58.20 °C, and at 56.5 °C PXM-407 showed its endothermic peak (Figure 6B).

DEX/HPβCD (1:4) binary complexes showed a significant drop in peak strength and decrease in the intensity of the endothermic peak of DEX. DEX in the binary complex was either amorphous or semi-crystalline. DSC thermographs showed that pure DEX was losing its fusion peak, indicating that all of the drug molecules were participating in complexation [56]. The melting points’ peaks were considerably reduced. A virtually flat region was achieved with polymers (PXM-188 and PXM-407), indicating the amorphousness and greater complexation with ternary inclusion complexes. The endothermic peak of DEX was not found in the DEX:HPβCD binary and also in ternary complexes. This may be due to the reduction in drug crystallinity [57]. DEX is in amorphous state or in a less crystal-like form in binary and ternary compounds. 

### 3.7. X-ray Diffractometry (XRD)

XRD is used to identify the presence of CD complexation in powder form. When a chemical (guest) interacts with a host (CD), changes in crystallinity can be detected by using this technique [58].

**Figure 7:**
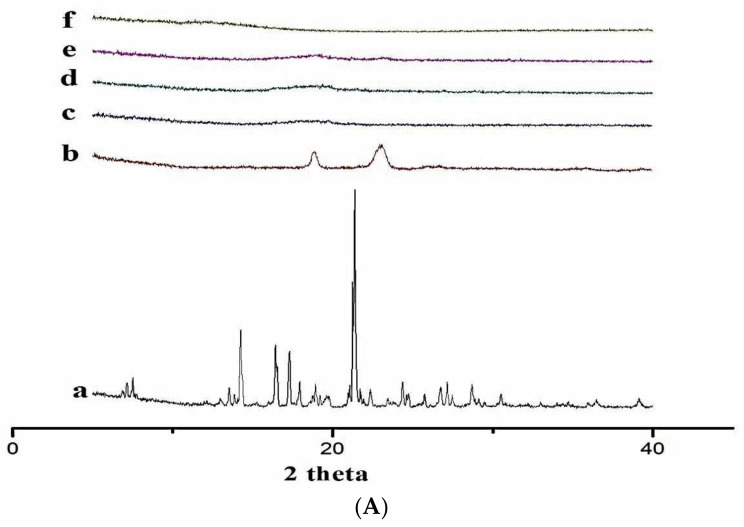

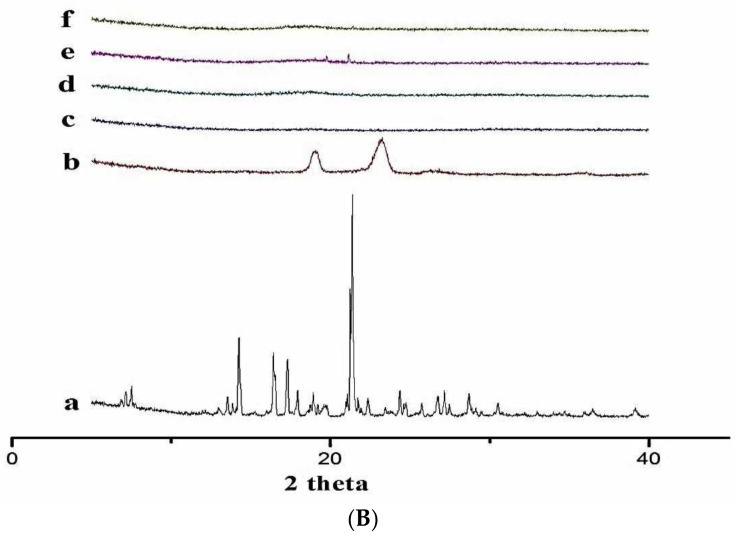
(**A**) XRD Diffractrograms of (a) DEX, (b) PXM-188, (c), 1:4 DEX:HPβCD, (d) 1:4 DEX:HPβCD:PXM-188 2.5%, (e) 1:4 DEX:HPβCD:PXM-188 5.0%, (f) 1:4 DEX:HPβCD:PXM-188 10% and (g) 1:4 DEX:HPβCD:PXM-188 20%. (**B**) XRD Diffractrograms of (a) DEX, (b) PXM-407, (c), 1:4 DEX:HPβCD, (d) 1:4 DEX:HPβCD:PXM-407 2.5%, (e) 1:4 DEX:HPβCD:PXM-407 5.0%, (f) 1:4 DEX:HPβCD:PXM-407 10% and (g) 1:4 DEX:HPβCD:PXM-407 20%.

Figure 7A,B shows the XRD diffractograms of DEX, hydrophilic polymers (PXM-188 and PXM-407), and binary and ternary complexes with these polymers. DEX generated sharp X-ray diffractograms, indicating its crystalline nature. PXM-188 produced the functional peaks at 19.17° and 23.33°, and pure PXM-407 X-ray diffractograms revealed distinct peaks at 18.89° and 23.09°.

The DEX–HPβCD inclusion complexes revealed the lower crystallinity of both compounds (drug and polymer) with flat peaks’ strength. The KN method could result in reducing the crystallinity in the inclusion complex. The reduced crystallinity and increased amorphousness result in low free Gibbs energy and high solubility [59]. Adsorption of polymer (amorphous) on surfaces of DEX causes the amorphousness. The smaller the particle size, the less sharp the X-ray diffractogram is expected to be, with several peaks disappearing [60]. In the case of the binary inclusion complex of DEX/HPβCD (1:4), both compounds (drug and polymer) were found to be amorphous, with higher flat peaks’ strength. It was illustrated that the binary complex showed less crystallinity or more amorphousness with high free Gibbs energy and solubility [61]. It is generally stated that the Gibbs energy is high in its amorphous state than its crystalline state (has low Gibbs energy), and because of this, ternary complexes have a more aqueous solubility and release rate as compared to DEX and binary complexes.

## 4. Conclusions

In this study, the binary-inclusion complex of DEX:HPβCD was prepared successfully. The samples showed a significant enhancement in their aqueous solubility and drug-release profile. The addition of hydrophilic polymers (PXM-188 and PXM-407) further increased the solubility and dissolution rate of the drug. The solid-state characterization results reported the formation of the complex with the presence of more agglomerated and amorphous DEX structure. Ternary complexes with PXM-188 presented a comparatively better increase in the solubility and dissolution of DEX.

## Figures and Tables

**Table 1:** Gibbs free energy of the DEX–CD complex in water at 25± 2 °C.

Concentration of HPβCD (mM/L)	ΔG^0^ (kcal mol^−1^)
5	−0.09
10	−0.12
15	−0.13
20	−0.15

**Table 2:** Stability constant and complexation efficiency results of binary and ternary inclusion complexes.

Inclusion Complexes	Stability Constant (M^−1^)	Complexation Efficiency
DEX:HPβCD	928.03 ± 0.1	0.65
DEX: HPβCD:PXM-188(1:4:2.5%)	1751.23 ± 0.2	0.12
DEX: HPβCD:PXM-188(1:4:05%)	3569.19 ± 1.1	0.49
DEX: HPβCD:PXM-188(1:4:10%)	4698.63 ± 0.5	2.5
DEX: HPβCD:PXM-188(1:4:20%)	5470.49 ± 0.4	3.29
DEX: HPβCD:PXM407(1:4:2.5%)	1573.20 ± 1.0	1.1
DEX: HPβCD:PXM-407(1:4:05%)	2610.45 ± 0.6	1.83
DEX: HPβCD:PXM-407(1:4:10%)	3854.24 ± 1.0	2.7
DEX: HPβCD:PXM-407(1:4:20%)	4825.60 ± 1.5	3.38

**Table 3:** Solubility data of DEX and binary and ternary inclusion complexes. Mean ± SD, *n* = 3.

DEX:HPβCD: Hydrophilic Polymers (*w/w*)	Solubility mg/mL
DEX	0.062 ± 0.01
DEX:HPCD1:1 (PT)	05.33 ± 0.63 *
DEX:HP-β-CD1:2 (PT)	07.83 ± 0.20 *
DEX:HP-β-CD1:4 (PT)	10.37 ± 0.38 *
DEX:HP-β-CD1:8 (PT)	11.55 ± 1.03 *
DEX:HP-β-CD 1:1 (KM)	6.98 ± 0.050 *
DEX:HP-β-CD 1:2 (KM)	10.90 ± 0.08 *
DEX:HP-β-CD 1:4 (KM)	17.13 ± 0.11 *
DEX:HP-β-CD 1:8 (KM)	18.23 ± 0.73 *
DEX:HP-β-CD1:1 (SE)	1.65 ± 0.150 *
DEX:HP-β-CD1:2 (SE)	1.89 ± 0.040 *
DEX:HP-β-CD1:4 (SE)	3.94 ± 0.000 *
DEX:HP-β-CD1:8 (SE)	5.04 ± 0.050 *
DEX: HPβCD:PXM-188 (1:4:2.5%)	24.83 ± 0.011 *^,α^
DEX: HPβCD:PXM-188 (1:4:5,0%)	30.86 ± 0.011 *^,α^
DEX: HPβCD:PXM-188 (1:4:10%)	40.54 ± 0.011 *^,α^
DEX: HPβCD:PXM-188 (1:4:20%)	42.66 ± 0.011 *^,α^
DEX: HPβCD:PXM-407 (1:4:2.5%)	19.50 ± 0.011 *^,α^
DEX: HPβCD:PXM-407 (1:4:5.0%)	20.48 ± 0.011 *^,α^
DEX: HPβCD:PXM-407 (1:4:10%)	33.34 ± 0.011 *^,α^
DEX: HPβCD:PXM-407 (1:4:20%)	34.88 ± 0.011 *^,α^

Note: * = comparison with pure drug, and α = comparison with binary inclusion complex (DEX:HPβCD at 1:4, prepared by KN method).

**Table 4:** Dissolution parameter of DEX and binary and ternary systems. Mean ± SD, *n* = 3.

DEX:HPβCD:POLYMER	DE60 (%)
DEX	38.76 ± 0.14
DEX:HPβCD1:1(PT)	62.76 ± 0.17 *
DEX:HPβCD1:2(PT)	62.90 ± 0.13 *
DEX:HPβCD1:4(PT)	62.82 ± 0.12 *
DEX:HPβCD1:1(KN)	63.21 ± 0.49 *
DEX:HPβCD1:2(KN)	63.56 ± 0.46 *
DEX:HPβCD1:4(KN)	62.98 ± 0.73 *
DEX:HPβCD1:1(SE)	63.93 ± 0.03 *
DEX:HPβCD1:2(SE)	63.35 ± 0.17 *
DEX:HPβCD1:4(SE)	63.70 ± 0.77 *
1: 4 (2.50% PMX-188)	65.71 ± 0.12 *^,α^
1: 4 (05.0% PMX-188)	66.01 ± 0.17 *^,α^
1: 4 (10.0% PMX-188)	65.85 ± 0.70 *^,α^
1: 4 (20.0% PMX-188)	66.18 ± 0.19 *^,α^
1: 4 (2.50% PMX-407)	65.27 ± 0.02 *^,α^
1: 4 (05.0% PMX-407)	65.80 ± 0.03 *^,α^
1: 4 (10.0% PMX-407)	65.52 ± 0.03 *^,α^
1: 4 (20.0% PMX-407)	64.83 ± 0.01 *^,α^

Note: * = comparison with pure drug, and α = comparison with binary inclusion complex (DEX:HPβCD at 1:4 prepared by KN method.

## Data Availability

Data is available on request.
